# Deciphering Microglial Contributions to Amyloid and Tau Pathology in Diverse Genetic Contexts

**DOI:** 10.1002/alz.092886

**Published:** 2025-01-03

**Authors:** Kristen D Onos, Kelly J Keezer, Bridget M Perkins, Anne Perkins, Adrian L Oblak, Gareth R Howell

**Affiliations:** ^1^ The Jackson Laboratory, Bar Harbor, ME USA; ^2^ Stark Neuroscience Research Institute, Indianapolis, IN USA

## Abstract

**Background:**

There is strong evidence that underlying genetics of an individual can significantly modify response to development of amyloid and tau pathology with age. Genome‐wide association studies indicate that variation in more than 25 genetic loci relevant to microglial biology are predicted to increase susceptibility to Alzheimer’s disease (AD). This work aims to understand how genetic context can impact microglia function, how these differences relate to AD pathology, and ultimately, cognitive decline.

**Method:**

To achieve this, we have introduced humanized amyloid‐β (with and without driver mutations) and *MAPT* sequences in two mouse strains, C57BL/6J (B6) and WSB/EiJ (WSB). In animals that carried amyloid driving mutations, we also performed injections of human‐derived tau at 6‐months and aged mice to 10‐months. Human brain tissue was obtained from the National Centralized Repository for Alzheimer’s Disease and Related Dementias (NCRAD) (ncrad.iu.edu). Immunohistochemistry was performed on mouse brain tissue to assess presence and degree of amyloid and tau pathology, differences in microglia and astrocyte activation and states, as well as neuronal and synaptic integrity. Measurements were also performed to assess degree of tau propagation. Plasma from animals was analyzed for a variety of biomarkers such as amyloid‐β, tau, neurofilament light, VEGFA and proinflammatory cytokines.

**Result:**

Data analysis is ongoing, however, the impact of the individual humanized alleles has been assessed. B6 and WSB mice that carry humanized amyloid‐β without mutations did not exhibit significant amyloid pathology at 10 months. However, B6 and WSB mice that carry the Swedish‐Arctic‐Austrian mutations do show significant parenchymal deposition starting at 6 months of age, and WSB exhibit mature cerebral amyloid angiopathy.

**Conclusion:**

We expect our strategy of incorporating humanized amyloid and tau into genetically diverse mouse strains will significantly improve the alignment of neuroimmune responses in mouse to human AD. This will give the AD field a new way to determine mechanisms, identifying critical genes and pathways in early in disease onset or progression. Furthermore, this may pave the way for the development of microglia‐based therapies to reduce or prevent tau propagation and spreading in AD and related dementias.

